# Visual acuity and electroretinography findings 3 ½ years after the first intravitreal injection of bevacizumab (Avastin) in aggressive posterior retinopathy of prematurity

**DOI:** 10.4103/0301-4738.73707

**Published:** 2011

**Authors:** Parag K Shah, Rodney J Morris, V Narendran, N Kalpana

**Affiliations:** Department of Pediatric Retina and Ocular Oncology, Aravind Eye Hospital and Postgraduate Institute of Ophthalmology, Coimbatore – 641 014, Tamilnadu, India

Dear Editor,

We reported the first case of intravitreal injection of bevacizumab (Avastin, Genentech, San Francisco, CA, USA) in aggressive posterior retinopathy of prematurity (APROP) in 2007.[1] Now we report visual acuity and electroretinography (ERG) findings of the same case, 3 ½ years after the initial injection.

**Figure 1:**
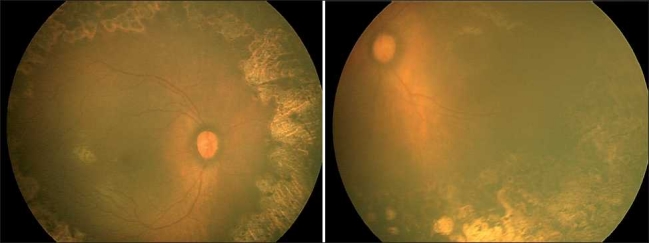
Retcam pictures showing regressed retinopathy of prematurity (ROP) in both eyes with peripheral laser scars extending till zone 1. Bevacizumab was given in left eye

**Figure 2:**
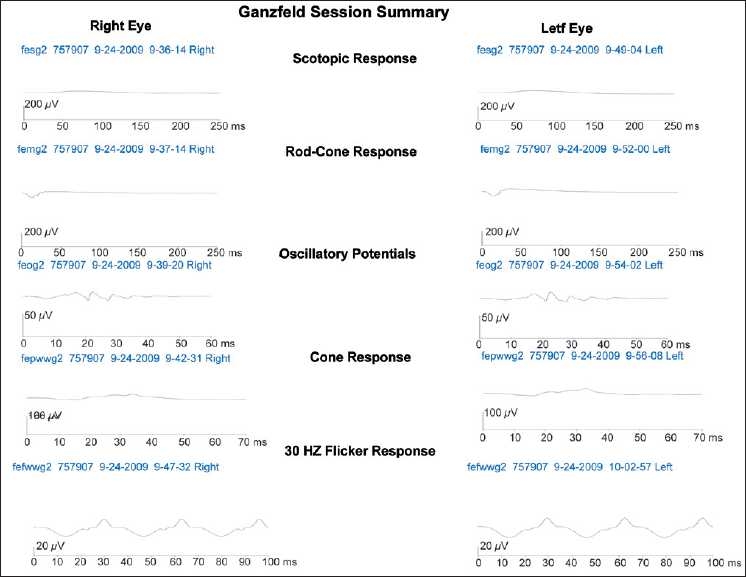
Ganzfeld session summary of both eyes showing symmetrical subnormal electroretinography (ERG) waves

A male born at 31 weeks of gestation and having a birth weight of 1170 g was diagnosed to have APROP. Immediate laser was done with almost 3,000 spots in each eye. One week after laser, both eyes developed anterior segment ischemia which was more severe in the left eye (LE). After taking an informed consent from the parents 0.03 ml bevacizumab (0.75 mg) was given intravitreally in LE. Right eye (RE) was managed conservatively. The features of anterior segment ischemia disappeared dramatically in LE after a single injection of bevacizumab, and at 10-month follow-up, fundus of both eyes showed regressed retinopathy of prematurity (ROP) with peripheral laser scars [Fig. 1]. Both eyes also developed lamellar cataracts which was more in LE.[1] The cataract in LE progressed further and retinoscopy increased to –21 diopters (D) in both the axes. The RE retinoscopy was –1.5 D. The axial length was 21.2 mm in LE and 18.7 mm in RE. Cataract surgery with primary posterior capsulorhexis, anterior vitrectomy, and posterior chamber intraocular lens implantation was done in LE. One month postsurgery his visual acuity was 20/180 in RE and 20/360 in LE on Teller’s acuity chart. He was advised occlusion of RE for 6 h/day for 4 months. At 3 ½ year follow-up, best corrected visual acuity was 20/30 in RE and only 20/500 in LE on Snellen acuity chart. There was no development delay seen in this child, and clinically the central nervous system, pulmonary, gastrointestinal, and renal systems were normal. Electro retinography (ERG) was done under general anesthesia. It showed subnormal and nearly extinguished B-wave in both eyes indicating extensive damage to rods and bipolar cells. The oscillatory potential response was present in both eyes, indicating near-normal vascularity. The cone response was noted as better than the rod response as indicated by the subnormal flicker ERG cone response. All the responses, although poor, were symmetrical in both eyes [Fig. 2].

ERG changes are seen in ROP irrespective of laser treatment. Postlaser treatment, both rods[2] and cones get affected, although rods are affected more than cones.[3] More severe the ROP, more involved are the photoreceptors.[3] Since our case had a severe form of APROP and extensively lasered, the ERG was expected to be subnormal. Although bevacizumab was given in LE, the ERG waves were comparable to RE. However the visual acuity was poor, compared to the other eye. This poor visual acuity could be a side effect of bevacizumab or may be due to amblyopia.

Our report suggests that intravitreal bevacizumab does not have an adverse effect on the ERG but could have on the final visual acuity. Thus, we urge caution in using it in premature babies till formal controlled studies with long-term follow-up are conducted to determine its potential safety in ROP.
